# Retraction: Macrophage Colony-Stimulating Factor Augments Tie2-Expressing Monocyte Differentiation, Angiogenic Function, and Recruitment in a Mouse Model of Breast Cancer

**DOI:** 10.1371/journal.pone.0235096

**Published:** 2020-06-17

**Authors:** The PLOS ONE Editors

After publication of this article [1], concerns were raised about results reported in Figure 1:

- In Figure 1B, several areas of the 0.1 ng/ml CSF1 panel appear similar to the corresponding areas in the 1 ng/ml CSF1 panel.
- In Figure 1C, several areas of the ANG2 panel appear similar to areas in the CSF1 + CSF1R Nab panel.

The authors noted that the original flow cytometry data (.fcs files) underlying the results in Figure 1 are no longer available. Quantitative data supporting the graphs in Figure 1 were provided, but without the original flow cytometry data we are unable to clarify the above concerns or confirm the reliability of the quantitative results.

In light of the unresolved concerns about Figure 1, the *PLOS ONE* Editors retract this article.

MAF, JLV, DD, LM, XM, CBM and TDE agreed with retraction but stand by the results and conclusions reported in this article. The other authors either did not reply directly or could not be reached.
